# Evaluation metrics and statistical tests for machine learning

**DOI:** 10.1038/s41598-024-56706-x

**Published:** 2024-03-13

**Authors:** Oona Rainio, Jarmo Teuho, Riku Klén

**Affiliations:** Turku PET Centre, University of Turku and Turku University Hospital, Turku, Finland

**Keywords:** Evaluation metrics, Machine learning, Medical images, Statistical testing, Statistics, Computer science

## Abstract

Research on different machine learning (ML) has become incredibly popular during the past few decades. However, for some researchers not familiar with statistics, it might be difficult to understand how to evaluate the performance of ML models and compare them with each other. Here, we introduce the most common evaluation metrics used for the typical supervised ML tasks including binary, multi-class, and multi-label classification, regression, image segmentation, object detection, and information retrieval. We explain how to choose a suitable statistical test for comparing models, how to obtain enough values of the metric for testing, and how to perform the test and interpret its results. We also present a few practical examples about comparing convolutional neural networks used to classify X-rays with different lung infections and detect cancer tumors in positron emission tomography images.

## Introduction

Due to our developed technology and access to huge amounts of digitized data, the number of different applications using machine learning (ML) has increased dramatically during the past few decades^1^. Whereas ML techniques initially included only statistical methods and simple algorithms^2^, ML is currently used for different purposes across the fields of engineering, medicine, public health, finance, politics, and natural sciences, both in academia and industry^3^. However, because of this immerse interdisciplinary interest, some of the new ML researchers might not have a good grasp of basic statistical concepts. This prompts need for ongoing education about the proper use of statistics and appropriate metrics for evaluation of performance of ML algorithms.

When new ML models are created, it is necessary to compare their performance to the already existing ones^4^. Evaluation serves two purposes: methods that do not perform well can be discarded, and the ones that seem promising can be further optimized. Also, especially in medicine, it is often useful to know whether an ML model outperforms an educated professional or not^5–7^. In supervised ML, we first divide our data for training and test sets, use the training data for training and validation of the model, predict all the instances of the test data, and compare the obtained predictions to the corresponding ground-truth values of the test set^8^. In this way, we can estimate whether the predictions of a new ML model are better than the predictions of a human or existing models in our test set.

Despite complexity of final applications, ML models typically consists of relatively simple sub-tasks, such as binary or multi-class classification and regression. In addition, a special image processing ML technique called a convolutional neural network (CNN) can be used to perform image segmentation^9^ and object detectors are used to find desired targets in images or video footage^10^. Depending on the task in question, there are certain choices of evaluation metrics that can be used to assess the performance of supervised ML models^11^. There are also established statistical testing practices, especially for metrics used in binary classification^8,12^. Nonetheless, the misuse of certain well-known tests, such as the paired t-test, is common^4^, and the required assumptions of the tests are often ignored^11^.

Our aim here is to introduce the most common metrics for binary and multi-class classification, regression, image segmentation, and object detection. We explain the basics of statistical testing and what tests should be used in different situations related to supervised ML. At the end, we also give three examples about comparing the performance of CNNs for classifying X-rays related to lung infections and performing image segmentation for positron emission tomography (PET) images.

## Different machine learning tasks

### Binary classification

In a binary classification task, the instances of data are typically predicted to be either positive or negative so that a positive label is interpreted as presence of illness, abnormality, or some other deviation while a negative instance does not differ from the baseline in this respect. Each predicted binary label has therefore four possible designations: a true positive (TP) is a correctly predicted positive outcome, a true negative (TN) is a correctly predicted negative outcome, a false positive (FP) is a negative instance predicted to be positive, and a false negative (FN) is a positive instance predicted to be negative^13^. A confusion matrix, here a $2\times 2$-matrix containing the counts of TP, TN, FP, and FN observations like Table 1, can be used to compute several metrics for the evaluation of the binary classifier.

**Table 1:** The confusion matrix of a modified U-Net CNN whose task was to classify 300 chest X-rays with COVID-19 in the test set as positive (pos.) and 300 X-rays from healthy patients as negative (neg.).

Predicted$\backslash$True class	Pos.	Neg.
Pos.	TP = 261	FP = 107
Neg.	FN = 39	TN = 193

The resulting values of different evaluation metrics can be found in Table 4.

The most commonly used evaluation metrics for binary classification are accuracy, sensitivity, specificity, and precision, which express the percentage of correctly classified instances in the set of all the instances, the truly positive instances, the truly negative instances, or the instances classified as positive, respectively. Sensitivity is commonly referred as recall^14^. They have the formulas

1 $$\begin{aligned} \begin{aligned} \mathrm{Acc.}&=\frac{\textrm{TP}+\textrm{TN}}{\textrm{TP}+\textrm{TN}+\textrm{FP}+\textrm{FN}}\in [0,1],\quad \mathrm{Sen.}=\mathrm{Rec.}=\frac{\textrm{TP}}{\textrm{TP}+\textrm{FN}}\in [0,1],\\ \mathrm{Spe.}&=\frac{\textrm{TN}}{\textrm{TN}+\textrm{FP}}\in [0,1],\quad \mathrm{Pre.}=\frac{\textrm{TP}}{\textrm{TP}+\textrm{FP}}\in [0,1], \end{aligned} \end{aligned}$$

where TP, TN, FP, and FN refer to the numbers of the predictions with these designations^13–16^. Especially in diagnostics, sensitivity or recall is also known as true positive rate^14^, specificity as true negative rate^16^, and precision as positive predictive value^17^. With the exception of accuracy, the aforementioned metrics are often used as pairs, such as precision and recall or sensitivity and specificity. It is noteworthy that sensitivity and specificity reveal more about the model than accuracy especially if the number of real positive and negative instances is very imbalanced.

There are also several other evaluation metrics like accuracy that depend on all the values of the confusion matrix: Youden’s index^18^, defined as $\mathrm{Sen.}+\mathrm{Spe.}-1$^15^, gives an equal weight to the accuracies within the positive and the negative instances, regardless of their numbers. The F1-score, defined as

$$\begin{aligned} \textrm{F1}=\frac{2\cdot \mathrm{Pre.}\cdot \mathrm{Rec.}}{\mathrm{Pre.}+\mathrm{Rec.}}\in [0,1], \end{aligned}$$

is a harmonic mean of precision and recall^19^. Cohen’s kappa ($\kappa$), defined as

2 $$\begin{aligned} \kappa =\frac{\mathrm{Acc.}-p_e}{1-p_e}\in (-\infty ,1]\quad \text {with}\quad p_e=\frac{(\textrm{TP}+\textrm{FN})(\textrm{TP}+\textrm{FP})+(\textrm{TN}+\textrm{FP})(\textrm{TN}+\textrm{FN})}{(\textrm{TP}+\textrm{TN}+\textrm{FP}+\textrm{FN})^2} \end{aligned}$$

compares how well the binary classifier performs compared to the randomized accuracy $p_e$^19^. It was originally introduced as a measurement for the degree of agreement between two observers in psychology^20^ but it can be applied to measure the agreement between the predicted and the real classes. Furthermore, Matthews’ correlation coefficient (MCC), defined as

3 $$\begin{aligned} \textrm{MCC}=\frac{\textrm{TN}\cdot \textrm{TP}-\textrm{FN}\cdot \textrm{FP}}{\sqrt{(\textrm{TP}+\textrm{FP})(\textrm{TP}+\textrm{FN})(\textrm{TN}+\textrm{FP})(\textrm{TN}+\textrm{FN})}}\in [-1,1], \end{aligned}$$

measures the correlation between the real and the predicted values of the instances^21^. This definition of MCC follows directly from that of Pearson’s correlation coefficient^22^.

To compute the values of the metrics above, the predictions of the test set by the model must be converted with some threshold if they are not already binary labels. The value of this threshold is often the default choice of 0.5 or the cut-point that gives highest accuracy or Youden’s index for the predictions of the training set. The threshold should be always chosen based on the predictions of the training set only because using the threshold that maximizes the accuracy of the predictions of the test set produces unrealistically good results.

**Figure 1:**
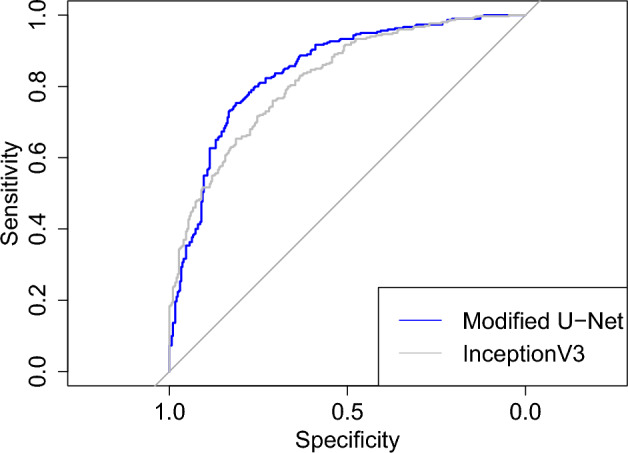
ROC curves computed from the binary predictions of a test set containing 300 chest X-rays with COVID-19 and 300 X-rays from healthy patients by the modified U-Net (in blue) and InceptionV3 (in gray), accompanied by a straight line equal to the theoretic ROC curve of a random binary classifier. The *x*-axis here uses sensitivity instead of the false positive rate but, since its values range from 1 to 0, the end result is a typical plot, not its reflection. The AUC values are 0.845 for the modified U-Net and 0.821 for InceptionV3. The values of other evaluation metrics are in Table 4.

However, if the numeric predictions before their conversion into binary are available, we can consider the receiver operating characteristic (ROC) curve. It is obtained by plotting sensitivity against the false positive rate (equal to 1 minus specificity) at all possible threshold values. As can be seen from Fig. 1, it follows that a ROC curve is always monotonically increasing function inside the unit square tied to the points (0, 0) and (1, 1) so that closer the ROC curve is to (0, 1) the better the predictions are^23^. The area under the ROC curve (AUC) is another possible evaluation metric with values in [0, 1] but, unlike for the metrics, its value does not depend on the choice of the threshold at all.

Alternatively, if we have *n* predictions $q_i\in (0,1]$ for binary labels $p_i\in \{0,1\}$, we can also compute their cross-entropy loss defined as

$$\begin{aligned} H(p,q)=-\sum ^n_{i=1}p_i\log (q_i). \end{aligned}$$

The cross-entropy loss is often used for training ML models as its values decrease as the differences between the predictions and the real binary labels diminish^24^.

### Multi-class classification

If the classification task is separating *n* instances between $k\ge 3$ different classes, we can present the results of the classifier by using a $k\times k$ confusion matrix as in Table 2. Its element $n_{ij}$ at the intersection of the *i*th and the *j*th column for $i,j=1,\ldots ,k$ is the number of instances from the *i*th classified to the *j*th class. The evaluation of this matrix uses same metrics that we introduced for binary classification.

**Table 2:** The confusion matrix of a modified U-Net CNN whose task was to separate 2800 chest X-rays of the set as into four different classes: negative (class 1), COVID-19 (class 2), pneumonia (class 3), or tuberculosis (class 4). We see, for instance, that the CNN classified most X-rays showing tuberculosis as COVID-19.

True$\backslash$Predicted class	1	2	3	4	Sum
1	120	7	9	4	$n_{1\cdot }$ = 140
2	15	116	3	6	$n_{2\cdot }$ = 140
3	12	13	115	0	$n_{3\cdot }$ = 140
4	2	96	4	38	$n_{4\cdot }$ = 140
Sum	$n_{\cdot 1}$ = 149	$n_{\cdot 2}$ = 232	$n_{\cdot 3}$ = 131	$n_{\cdot 4}$ = 48	*n* = 560

Based on this confusion matrix, the CNN has accuracy of 0.847, sensitivity of 0.695, specificity of 0.898, macro-average precision of 0.744, micro-average precision of 0.695, Youden’s index of 0.593, macro-average F1-score of 0.677, micro-average F1-score of 0.695, $\kappa$ of 0.598, and MCC of 0.616.

Firstly, there are two simple ways to obtain the values for all the evaluation metrics except AUC introduced with the previous section. We need to create a unique $2\times 2$ confusion matrix for each of the *k* classes:

$$\begin{aligned} \textrm{TP}_i=n_{ii},\quad \textrm{TN}_i=\sum_{j\neq i}\sum _{h\ne i}n_{jh},\quad \textrm{FN}_i=\sum _{j\ne i}n_{ij},\quad \textrm{FP}_i=\sum _{j\ne i}n_{ji},\quad i=1,\ldots ,k. \end{aligned}$$

In a process called macro-averaging, we calculate the value of the metric separately for each class $i=1,\ldots ,k$ by using the numbers $\textrm{TP}_i$, $\textrm{TN}_i$, $\textrm{FN}_i$, and $\textrm{FP}_i$ defined as above and then consider the mean value of the *k* resulting values of the metric. Alternatively, in micro-averaging, we compute the value of the evaluation metric from the sums $\sum ^k_{i=1}\textrm{TP}_i$, $\sum ^k_{i=1}\textrm{TN}_i$, $\sum ^k_{i=1}\textrm{FN}_i$, and $\sum ^k_{i=1}\textrm{FP}_i$. Out of these procedures, macro-averaging gives equal weight to each class regardless of their size whereas micro-averaging gives equal weight to each instance and is therefore easily dominated by larger classes^25^. However, if each class should contain equally many instances as in the situation of in Table 2, both micro- and macro-averaging yield same values for accuracy, sensitivity, specificity, and Youden’s index.

Cohen’s $\kappa$ and MCC have also own definitions specially designed for the multi-class classification: Cohen’s $\kappa$ can be written as

$$\begin{aligned} \kappa =\frac{p_0-p_e}{1-p_e}\quad \text {with}\quad p_0=\frac{1}{n}\sum ^k_{i=1}n_{ii},\quad p_e=\frac{1}{n^2}\sum ^k_{i=1}n_{i\cdot }n_{\cdot i}, \end{aligned}$$

where $n_{i\cdot }=\sum ^k_{j=1}n_{ij}$, $n_{\cdot i}=\sum ^k_{j=1}n_{ji}$, and $n=\sum ^k_{i=1}\sum ^k_{j=1}n_{ij}$^26^. Similarly, MCC can be computed from a general $k\times k$ confusion matrix with the formula^27^

$$\begin{aligned} \textrm{MMC}=\frac{n\sum ^k_{i=1}n_{ii}-\sum ^k_{i=1}n_{i\cdot }n_{\cdot i}}{\sqrt{(n^2-\sum ^k_{i=1}n^2_{i\cdot })(n^2-\sum ^k_{i=1}n^2_{\cdot i})}}. \end{aligned}$$

In the special case $k=2$, we obtain the same formulas for Cohen’s $\kappa$ and MCC as in (2) and (3)^22^.

### Multi-label classification

Multi-label classification is a generalized version of multi-class classification with nonexclusive class labels. Instead of dividing the data instances between several classes, the aim is to find all the class labels that apply out of $k\ge 2$ possible labels. For each *n* instances, the model returns a binary vector $y^{(i)}$, $i=1,\ldots ,n$, whose *j*th element is 1 if the *j*th label is present and otherwise 0 for all $j=1,\ldots ,k$. A possible metric for evaluation is the Hamming loss, defined as

$$\begin{aligned} \frac{1}{nk}\sum ^n_{i=1}\sum ^k_{j=1}|x^{(i)}_j-y^{(i)}_j|\in [0,1], \end{aligned}$$

where $x^{(i)}_j$ is the real value of the *j*th element in the binary vector of the *i*th data instance and $y^{(i)}_j$ is the corresponding predicted value. The smaller the Hamming loss is, the better the model is. Alternatively, we can compute for instance the micro- or macro-average accuracy, precision, or recall for the vectors $y^{(i)}$, $i=1,\ldots ,n$^28^.

### Regression

In a regression problem, a model is used predict instances whose values are real numbers rather than categorical. This is the case when predicting, for instance, height, stock prices, voter turnout, or rainfall amount. Here, we denote the real value of the *i*th instance in a test set of *n* instances by $x_i$ and its predicted value by $y_i$ for $i=1,\ldots ,n$.

One way to evaluate the model is to measure correlation between the real and the predicted values^12^. The most well-known method for this is Pearson’s correlation coefficient, defined as

4 $$\begin{aligned} r=\frac{\sum ^n_{i=1}(x_i-\overline{x})(y_i-\overline{y})}{\sqrt{\sum ^n_{i=1}(x_i-\overline{x})^2\sum ^n_{i=1}(y_i-\overline{y})^2}}\in [-1,1], \end{aligned}$$

where $\overline{x}$ and $\overline{y}$ denote the mean values of the vectors $(x_1,\ldots ,x_n)$ and $(y_1,\ldots ,y_n)$, respectively^29^. However, Pearson’s correlation coefficient is designed for measuring correlation between variables whose marginal distributions are assumed to be normal. Because of this, Spearman’s correlation coefficient $r_s$ might be a better evaluation metric when the real values $x_i$ are not even approximately normally distributed. Spearman’s correlation coefficient is obtained by first converting the observations $x_i$ and $y_i$, $i=1,\ldots ,n$, into their ranks and then computing Pearson’s correlation coefficient of these ranks^29^.

Another way to evaluate the model is to use some error measurement, such as mean absolute error (MAE) $\sum ^n_{i=1}|x_i-y_i|$ or mean squared error (MSE) $\sum ^n_{i=1}(x_i-y_i)^2$^12^. The difference between MSE and MAE is that MSE punishes more for large errors^12^. Naturally, the smaller the error measurement is, the better the model performs.

### Image segmentation

**Figure 2:**
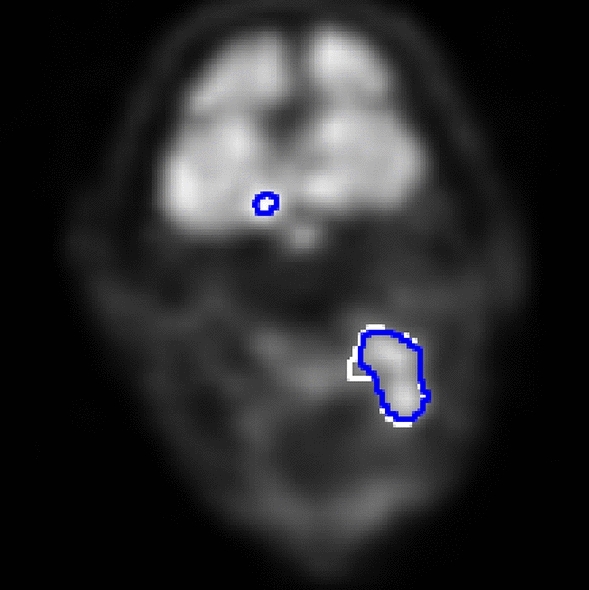
The binary tumor mask predicted by U-Net CNN with maximum dimensionality of 128 (in blue) and the ground-truth tumor mask drawn by a physician (in white) for one transaxial slice from a PET image of a head and neck cancer patient. The image is 128 $\times$ 128 pixels and the predicted segmentation mask contains 181 TP pixels, 16156 TN pixels, 17 FP pixels, and 30 FN pixels. This gives us Dice of 0.885, IoU of 0.794, and overall pixel accuracy of 0.997.

Image segmentation is a process of dividing images into regions of pixels or, in case of three-dimensional (3D) images, voxels, so that different objects and their boundaries can be located. In practice, this means converting a matrix of the same size as an image into a segmentation mask whose each point tells the class of the corresponding point in the image. In binary image segmentation, the desired output is a binary mask with positive elements coded as 1s and negative elements as 0s but we can also perform multiclass image segmentation called semantic segmentation by using more integers to signify different classes. An example of binary tumor segmentation can be seen in Fig. 2.

One of the possible evaluation metric for an image segmentation masks is accuracy. In case of binary segmentation, we could simply count the number of TP, TN, FN, and FP pixels and calculate the accuracy as in (1). However, the issue with this approach is that the number of positive pixels is typically very small compared to the number of negative pixels: For instance, if we try perform tumor segmentation for medical images of the body, the positive targets, while incredibly important, have minimal volume compared to the background and they might not even be present in some images. Because of this, the value of accuracy can be very high even in the cases where the model does not find the positive object as long as the majority of negative pixels is correct.

Consequently, the results of binary segmentation are often evaluated with a metric that ignores the TN points. Instead, we concentrate on evaluating the similarity of the predicted positive segment given by a CNN and the ground-truth positive segment annotated by a human. For this purpose, we can use the Sørensen–Dice similarity coefficient^30,31^, also known as the Dice score, defined for two sets *X* and *Y* as

5 $$\begin{aligned} D=\frac{2|X\cap Y|}{|X|+|Y|}\in [0,1], \end{aligned}$$

where |*S*| denotes the number of pixels or voxels in the set *S*^32^. This definition can be equivalently written as

$$\begin{aligned} D=\frac{2\cdot \textrm{TP}}{2\cdot \textrm{TP}+\textrm{FP}+\textrm{FN}}\in [0,1] \end{aligned}$$

by using the elements of the confusion matrix from the binary predictions of the points^32^. A very similar alternative to Dice score is the Jaccard similarity coefficient^33^, which is also known as the Jaccard index or Intersection over Union (IoU), and defined as

$$\begin{aligned} \textrm{IoU}=\frac{|X\cap Y|}{|X\cup Y|}\in [0,1] \end{aligned}$$

for the sets *X* and *Y*, and

6 $$\begin{aligned} \textrm{IoU}=\frac{\textrm{TP}}{\textrm{TP}+\textrm{FP}+\textrm{FN}}\in [0,1] \end{aligned}$$

for the elements of the confusion matrix^32^. The equality $\textrm{IoU}=D/(2-D)$ holds trivially between the IoU and the Dice score^32^.

There are also metrics specially designed for 3D segmentation, as this is common task for medical tomography images. The surface of the point set *X*, denoted by $\partial X$, is the set of all voxels in *X* for which at least one of the 18 or the 26 neighbour voxels is does not belong in *X*. As an alternative to the typical Dice score, the surface Dice similarity coefficient (SDSC) can be computed by replacing *X* and *Y* with their surfaces $\partial X$ and $\partial Y$ in (5). Let *d*(*x*, *y*) be the Euclidean distance between two voxels *x* and *y*, and define $d(x,Y)=\min _{y\in \partial Y}d(x,y)$ for the set *Y*. The average symmetric surface distance (ASD) between sets *X* and *Y* can now be defined as

$$\begin{aligned} \textrm{ASD}=\frac{1}{|X|+|Y|}\left( \sum _{x\in \partial X}d(x,Y)+\sum _{y\in \partial Y}d(y,X)\right) . \end{aligned}$$

The Hausdorff distance is $\textrm{hd}(X,Y)=\max _{x\in X}d(x,Y)$ and its symmetric version, also known as the maximum symmetric surface distance, is $\textrm{HD}(X,Y)=\max \{\textrm{hd}(X,Y),\textrm{hd}(Y,X)\}$. The symmetric volume difference (SVD) is a Dice-based error metric defined as $\textrm{SVD}=1-D$ and the volumetric overlap error (VOE) is the corresponding error measure derived from IoU, $\textrm{VOE}=1-\textrm{IoU}$. The model performance is considered better with smaller surface distances and errors terms^34^.

The results of multi-class semantic segmentation are typically evaluated by using mean Dice or IoU values, either as the mean of all within-class scores in a single image or the class-specific means of several images. The similarity of two semantic segmentation masks or any two can be also evaluated with structural similarity index measure (SSIM). If *u* and *v* are two image matrices with means $\overline{u}$ and $\overline{v}$, variances $s_u$ and $s_v$, and covariance $s_{u,v}$, then we have

$$\begin{aligned} \textrm{SSIM}(u,v)=\frac{2\overline{u}\overline{v}+c_1}{\overline{u}^2+\overline{u}^2+c_1}\frac{2s_us_v+c_2}{s_u^2+s_v^2+c_2}\in [-1,1] \end{aligned}$$

for constants $c_1$ and $c_2$ depending on pixel values^35^. The SSIM is typically computed by using the formula above within several kernels or windows of the images. The values of SSIM are interpreted as those correlation: 1 for perfect similarity, 0 for no association, and $-1$ for perfect opposites.

### Object detection

Another similar tasks related to image processing is object detection, in which we find bounding boxes around each object in the image and classify them into different classes. A good object detector is capable of finding all the objects in an image without producing any false observations, placing the bounding boxes as close their correct locations as possible, and also classifying all the found objects correctly. Due to the diversity in these subtasks, evaluation of object detectors is slightly more complicated than it is for the other models introduced.

To evaluate the results of object detection, we must start by counting how many objects of a specific class were found. This quickly leads to the question how to decide how close a predicted bounding box needs to be a ground-truth box so that we can interpret the object as found. The common criteria here is IoU defined as in (6): The prediction is only considered a match of a ground-truth box if the IoU value of the two boxes exceeds a certain threshold value, often 0.5. If there are several predicted boxes producing an IoU high enough with the same ground-truth box, only the best one in terms of IoU is considered a match to the ground-truth box while all the others are FP observations. Namely, FP is here the number of predicted boxes without a matching ground-truth box while TP is the number of the predictions that match a ground-truth box of the same class and FN is the number of ground-truth boxes without a matching prediction^10^.

With the TP, FP, and FN numbers of the specific class, we can compute precision and recall as in (1). Since an object detector outputs a confidence for every bounding box expressing how confident the model is about the prediction, we can remove the predictions below a threshold of confidence. Changing this threshold affects TP, FP, and FN numbers and therefore also precision and recall. The precision-recall curve (PRC) can be obtained by plotting precision against recall at all possible thresholds of confidence. After that, we can compute average precision (AP) as the area under the PRC. The whole model is evaluated by computing mean average precision (mAP) as the mean value of the APs in all the different classes. We often consider mAP@0.5 which is computed by using the IoU threshold 0.5 to define a match but just as well we could compute mAP@0.75 or mAP@0.9, or mAP@[0.5:0.95] which is the the mean value of mAP@0.5, mAP@0.55, $\ldots$, mAP@0.95. The metric mAP@0.9 is more strict than mAP@0.5 given it requires greater overlap for the potential matches and is therefore suitable for situations where the predicted bounding box locations need to be very exact^10^.

### Information retrieval

Information search and retrieval is a significant task in ML research. The ability to retrieve only relevant results from large image- or text-based databases is crucial for these databases to be actually useful. Search engines and other information retrievals models can be evaluated by using precision and recall to describe the percentage of relevant retrieved documents among either search results or all the relevant documents. If we have *K* results $d_1,\ldots ,d_K$ ordered by estimated relevance from the database *D* and each document *d* is either relevant ($\textrm{rel}(d)=1$) or not ($\textrm{rel}(d)=0$), we can compute precision of the first *k* retrieved documents as P@$k=\sum ^k_{i=1}\textrm{rel}(d_i)/k$, for $k=1,\ldots ,K$ and then define AP as^36^

$$\begin{aligned} \textrm{AP}=\frac{\sum ^K_{k=1}\textrm{rel}(d_k)\cdot \mathrm{P@}k}{\sum _{d\in D}\textrm{rel}(d)}. \end{aligned}$$

The mAP is obtained by a mean value of AP across different topics or search queries^36^. If results have more classes than just relevant and non-relevant, discounted cumulative gain (DCG) of *k* first results can be defined as

$$\begin{aligned} \textrm{DCG}=\sum ^k_{i=1}\frac{G(i)}{\log _2(i+1)}, \end{aligned}$$

where *G*(*i*) is a numerical value presenting the gain of the *i*th result^37^. For instance, the values 10, 7, 3, 0.5, and 0 are often used for perfect, excellent, good, fair, and bad results, respectively^37^. If there are several search queries to be evaluated, mean DCG can be used.

## Statistical tests

The motivation behind statistical tests is often to find out whether there is a significant difference between two different populations within respect of some specific property. We can collect smaller data sets from the populations and use them to compute values of the numeric quantity representing the feature of interest. Since there is nearly always at least slight difference between these values, the relevant question is whether this difference is great enough to be considered as an actual evidence of an underlying dissimilarity between the populations or if it is just a result of random variation.

The process of statistical testing is relatively simple: We formulate a null hypothesis $H_0$ according to which there is no real difference, choose some level of significance $\alpha \in (0,1)$, and define a suitable test statistic *Z* with a known probability distribution $P(Z|H_0)$ under the null hypothesis. We then use this distribution to compute the probability of obtaining at least as extreme value for the statistic *Z* than the one value *z* already observed. If the resulting probability $p=2\min \{P(Z\le z|H_0),P(Z\ge z|H_0)\}$, called *p* value, is less than $\alpha$, then the null hypothesis is rejected and the difference is considered statistically significant. We make a type I error when rejecting a true null hypothesis, and a type II error is accepting a false null hypothesis. We can control the probability of a type I error as its is equal to $\alpha$. We could also use $\alpha$ to compute the critical values for the statistic for accepting or rejecting the null hypothesis instead of using a *p* value. However, in this paper, all the test functions in Python^38^ and R^39^ mentioned return a *p* value. We use $\alpha =0.05$ as the level of significance in our examples.

**Figure 3:**
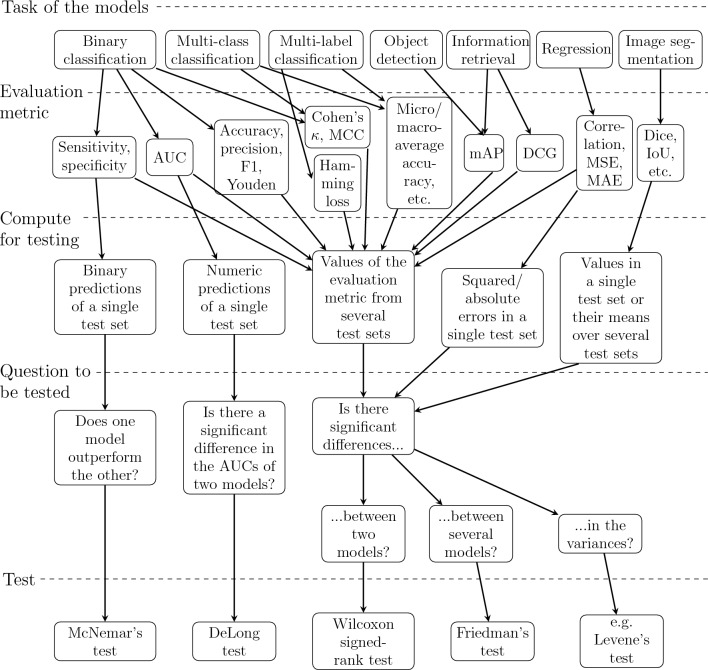
The possible tasks for a model, their evaluation metrics, the values of the evaluation metric that must be computed for each model before statistical testing, the potential questions a statistical test could answer in the situation, and the suitable test.

When comparing performance of two or more models, it is often necessary to perform the tests for multiple times depending on the evaluation metric and the statistical test used. For instance, while we can compute Dice score of every predicted segmentation mask in the test set, we only obtain one value of accuracy from the predictions of the whole test set after binary classification and as well as one value of MSE after regression. If we want to compare regression models, we can test squared errors instead of their mean and, in case of binary classification, there are tests that are based on the predictions of a single test set. In other cases, we have to evaluate our models on several test sets to obtain enough values from other evaluation metrics for statistical testing. The required values of an evaluation metric for a certain statistical test are summarized in the flowchart of Fig. 3.

While the test sets should ideally come from fully different data sets, sometimes our only option is to use a resampling procedure to create multiple test sets from the same data. In practice, we must re-initialize, train, and test the models for several times and save the values of the evaluation metrics from the predictions of the test set on each iteration round. We should use same training and test set for all the models on the same iteration round but vary them between the rounds because, otherwise, our conclusions about a potential difference between the models might be misled by some unknown factor in these specific data sets. Researchers commonly use here *k*-fold cross-validation, in which the data is divided into *k* similarly sized folds and, during *k* iteration rounds, each fold is the test set exactly once while the other $k-1$ form the training data^12^. Alternatively, we can perform repeated cross-validation that has a few re-runs of each potential test set^12^. However, it should be taken into account that resampling methods do not produce independent values for the evaluation metrics and might lead to underestimating the variance of the test statistic, causing biased results^12^.

### Testing for a significant difference in any evaluation metric

Regardless of whether the values of the evaluation metric come from a single test set or several test sets on different iteration rounds, the values of the metric for the two models are based on the same instances and therefore paired. Many researchers therefore check which of the models gives a higher mean and then use a paired t-test to test if the difference in the mean is significant^4^. The null hypothesis of the paired t-test is that the mean of the differences in the matched pairs is equal to 0^40^, and this test can be performed with the function ttest_rel in the package scipy.stats^41^ in Python or t.test(x,y,paired=TRUE) in the base package stats in R. There are also such newer variations of the t-test that are specially designed to repeated cross-validation^11^. However, the t-test is not recommended for this situation because it is strongly affected by outliers^4^ and not valid when resampled test sets are used^12^.

Another possible test is a sign test. If two models are evaluated by using *N* test sets and there is no difference between them, then each of them should produce a better value for the evaluation metric *N*/2 times^4^. Thus, the number of times where the first model is better than the second follows a binomial distribution and, for a greater number of *N*, a normal distribution with a mean *N*/2 and standard deviation $\sqrt{N}/2$^11^. We can therefore apply the sign test to test whether one of the models outperforms the other with respect to the chosen evaluation metric in a statistically significant way. However, the sign test has a very weak power for detecting significant differences^4^.

The best alternative for this situation is the Wilcoxon signed-rank test instead^4^. It is a non-parametric test for the null hypothesis that the median of the differences in the matched pairs is equal to 0^42^. This test has the test statistic

$$\begin{aligned} T=\min \{R^+,R^-\},\quad \text {where}\quad R^+=\sum _{d_i>0}\textrm{rank}(|d_i|),\quad R^-=\sum _{d_i<0}\textrm{rank}(|d_i|), \end{aligned}$$

and $\textrm{rank}(|d_i|)$, $i=1,\ldots ,n$, denote the differences $d_i$ in the *n* matched pairs ranked by their absolute values^43^. The *T*-statistic can be examined directly by using its own critical values or, for large values of *n*, utilizing the statistic

$$\begin{aligned} z=\frac{T-n(n+1)/4}{\sqrt{n(n+1)(2n+1)/24}}, \end{aligned}$$

which follows the normal distribution under the null hypothesis^4^. The Wilcoxon signed-rank test can be performed with wilcoxon in scipy.stats in Python or wilcox.test(x,y, paired=TRUE) in stats in R.

### Test for comparing several models

As explained above, we can use Wilcoxon signed-rank test to estimate whether the differences between two models are significant with respect to any evaluation metric, but this test is not ideal when comparing several models. Namely, while we can repeat Wilcoxon tests between each pair of models, the risk of type I error increases with multiple comparisons. Adjusting the level of significance by Bonferroni correction has been suggested as a solution^44^ but it is overly radical^4^.

Instead, the better approach in a situation where we have *K* models evaluated in *J* data sets is to perform Friedman’s test^4^. The average rank of the *k*th model, $k=1,\ldots ,K$, is $\overline{R}_k=\sum ^J_{j=1}r^j_k/J$ where $r^j_k$ is the rank of the *j*th value of the evaluation metric for the *k*th model^4^. The test statistic can be now written as

$$\begin{aligned} \chi ^2_F=\frac{12J}{K(K+1)}\left( \sum ^K_{k=1}\overline{R}^2_k-\frac{K(K+1)^2}{4}\right) \end{aligned}$$

or, as noted by Iman and Davenport^45^, as^4^

$$\begin{aligned} F_{ID}=\frac{(J-1)\chi ^2_F}{J(K-1)-\chi ^2_F}. \end{aligned}$$

Out of the two statistics, $\chi ^2_F$ is overly conservative and $F_{ID}$ is therefore recommended^4^. Under the null hypothesis, $\chi ^2_F$ follows the $\chi ^2$-distribution with $K-1$ degrees of freedom and $F_{ID}$ follows the *F*-distribution with $K-1$ and $(K-1)(J-1)$ degrees of freedom^4^. Friedman’s test can be performed with friedmanchisquare in scipy.stats in Python or friedman.test in stats in R, but both of these functions are based on the statistic $\chi ^2_F$ and therefore are not reliable for small values of *J*. However, if *J* is small, we can use a few separate Wilcoxon signed-rank tests instead.

### Tests for binary classification of a single test set

There are also such tests for comparison of two classifiers which only require their predictions from a single iteration round. McNemar’s test is a common non-parametric test that only requires two numbers and is typically used to compare either sensitivity or specificity of two classifiers^46^. To find out whether there is a significant difference in the sensitivity of the classifiers, let *b* be the number of positive instances in the test set misclassified as FN by the first classifier but not by the second classifier and *c* similarly the number of positive instances misclassified as FN by the second classifier but not by the first classifier. To study specificity, count the numbers *b* and *c* by using FP misclassifications among the negative instances. Comparing accuracy by counting errors among both positive and negative sets is not recommended^47^. If there is no significant difference in the performance of the two classifiers, the test statistic

$$\begin{aligned} \frac{(|b-c|-1)^2}{b+c} \end{aligned}$$

follows the $\chi ^2$-distribution with 1 degree of freedom for $b+c\ge 20$ and a binomial distribution otherwise^11^. This test can be performed with mcnemar in statsmodels.stats.contingency_tables^48^ in Python or mcnemar.test in stats in R.

We can also use the DeLong test to see whether there is a statistically significant different between the AUCs of two binary classifiers. Namely, DeLong et al.^49^ noticed that the Mann-Whitney statistic can be used as an estimate of an AUC and the theory of generalized U-statistic can be applied to compare two AUCs. The Mann-Whitney two-sample statistic for AUC can be written as

$$\begin{aligned} \hat{\theta }=\frac{1}{mn}\sum ^m_{i=1}\sum ^n_{j=1}\Psi (Y_{i1},Y_{j0})\quad \text {with}\quad \Psi (Y_{i1},Y_{j0})={\left\{ \begin{array}{ll} 1&{} \quad \text {if }Y_{i1}>Y_{j0},\\ 1/2&{} \quad \text {if }Y_{i1}=Y_{j0},\\ 0&{} \quad \text {if }Y_{i1}<Y_{j0}, \end{array}\right. } \end{aligned}$$

where *m* is the number of truly positive instances, *n* is the number of the number of truly negative instances, $Y_{i1}$ is the numeric prediction of the *i*th positive instance before it was converted into binary and, similarly, $Y_{j0}$ is the numeric prediction of the *j*th negative instance^50^. Let $\hat{\theta }_1$ be the estimate above for the AUC of the first classifier and $\hat{\theta }_2$ the same for the second classifier. The DeLong test estimates their variance and covariance (see e.g.^51^ for the exact formulas) and then uses the statistic

$$\begin{aligned} Z_D=\frac{\hat{\theta }_1-\hat{\theta }_2}{\sqrt{{\textrm{Var}}(\hat{\theta }_1)+{\textrm{Var}}(\hat{\theta }_2)-2{\textrm{Cov}}(\hat{\theta }_1,\hat{\theta }_2)}}, \end{aligned}$$

which follows the normal distribution under the null hypothesis due to the properties of the known U-statistic^51^. The DeLong test can be performed with roc.test(x,y,method= ’delong’) in the package pROC^52^ in R.

### Tests for comparing variance

Another important factor when comparing the performance of models is the amount of variance they produce. A model that consistently obtains high values in some evaluation metric is better than a model whose performance varies greatly on different iteration rounds. However, it must be taken into careful consideration here how the multiple values of the evaluation metric are obtained before considering their variance. For instance, if we use repeated cross-validation, we will not obtain a realistic estimate how the performance of a model would vary over different data sets.

We can use the F-test of equality of variances to test the null hypothesis according to which two populations have equal variances. The test statistic is $F=S^2_1/S^2_2$ where $S^2_1$ and $S^2_2$ are the sample variations in the values produced by the two models for the evaluation metric, and this F-statistic follows the F-distribution with $n-1$ and $n-1$ degrees of freedom under the null hypothesis^53^.

However, the use of the F-test is not recommend for non-normally distributed values and this is often the case when comparing evaluation metrics: For instance, if the model has a median accuracy of 90% but a high amount of variation between different test sets, it is likely that the distribution of accuracy is left-skewed as the accuracy is limited on [0, 1] by its definition. The normality can be tested here with the Shapiro–Wilk test^54^ (shapiro in the package scipy.stats and shapiro.test in the package stats in R). If the data is not normally distributed, the possible alternatives for the F-test include Barlett’s test^55^ (bartlett in scipy.stats in Python and bartlett.test in stats in R) and Levene’s test^56^ (levene in scipy.stats in Python and leveneTest in the package car^57^ in R).

### Comparison to a human

In ML research, it is often of interest if a specific ML model performs better than a human. Especially, in a medical field, it is useful to estimate the difference between the tumor masks predicted by a CNN differ and those drawn by a physician by taking into account how much difference there would be if the same masks were drawn by two different physicians. For this purpose, we can use statistical testing to compare the results of an ML model and a human in terms of a relevant evaluation metric as we would compare the performance of two models. However, there might be some cases where this comparison is not possible: A human is not able to go through very large amounts of data, at least not fast, and, while we can always re-initialize the model between different rounds of repeated cross-validation, a human will not forget their earlier decisions. Because of this, statistical comparison between an ML model and a human is often limited to using McNemar’s test or the DeLong test to compare classifications in a single test set or the Wilcoxon signed-rank test to compare segmentation masks in terms of Dice and IoU values for a reasonable number of images.

## Examples

### Software requirements

The CNNs were coded in Python (version: 3.9.9)^38^ with packages TensorFlow (version: 2.7.0)^58^ and Keras (version: 2.7.0)^59^. Most of the test were preformed in Python with scipy (version: 1.7.3)^41^ or statsmodels (version: 0.14.0)^48^. The DeLong test was performed and Fig. 1 was plotted with pROC (version: 1.18.5)^52^ in R (version: 3.4.1)^39^. The images of the third data set had been studied with Carimas (version: 2.10)^60^, which was also used to draw their binary masks.

### Data

We use three data sets consisting of two-dimensional grayscale images converted into the size of 128 $\times$ 128 pixels. The first data set contains 3000 chest X-rays of COVID-19 patients and 3000 chest X-rays of healthy patients chosen from COVID-19 Radiography Database^61,62^. The second data set has 700 chest X-rays of healthy patients and 700 chest X-rays of COVID-19 patients from COVID-19 Radiography Database, 700 chest X-rays of patients with pneumonia from Chest X-Ray Images (Pneumonia)^63^, and 700 chest X-rays of tuberculosis patients from Tuberculosis (TB) Chest X-ray Database^64^. The third data set has a total of 962 two-dimensional transaxial image slices from the PET images of 89 head and neck squamous cell carcinoma patients. The patients were imaged with $^{18}$F-fluorodeoxyglucose tracer in Turku PET Centre, Turku, Finland, during years 2014–2022. More details about the imaging can be found in^65,66^. Each of the slices has also a ground-truth binary segmentation mask showing pixels depicting cancerous tissue as positive and the rest as negative, and they were chosen so that they have at least 6 positive pixels. All the cancer patients were at least 18 years of age, gave informed consent to the research use of their data, and the research from their data was approved by Ethics Committee of the Hospital District of Southwest Finland. All research was performed in accordance with the Declaration of Helsinki.

### Convolutional neural networks

In both binary and multi-class classification, we use a CNN that has U-Net architecture by Ronneberger et al.^67^ modified for classification^65^ and a ready-built CNN called InceptionV3 available in Keras. For binary segmentation, we use two U-Nets, a shallower of which has 64 as maximum dimensionality of a Conv2D layer and a deeper of which has 128. They were also used in^66,68^. We use stochastic gradient descent as an optimizer for the classification CNN and Adam for the segmentation CNNs. The classification CNNs are trained on 10 epochs and the segmentation CNNs on 50. The learning rate of 0.001 and, during training, 30% of the training data is used for validation. After training the CNNs for binary classification, we predict both training and test sets and use the threshold giving the maximal Youden’s index in the training set as a threshold for converting the numeric predictions of the test set into binary labels. We similarly convert the output after binary segmentation by using the threshold that produces the highest median Dice in the training set. For the multi-class classification, we obtain directly class labels by using the maximum elements of one-hot encoding.

### Our experiments

We first compare the performance of the modified U-Net and InceptionV3 in binary classification by using our first data set of COVID-19 and negative X-rays with fivefold cross-validation. We compute all the possible evaluation metrics from our single test set and use McNemar’s test for sensitivity and specificity and DeLong test for AUC. Then we compare the modified U-Net and InceptionV3 in multi-class classification with repeated fivefold cross-validation (5 re-runs of each test set). We save the values of micro- and macro-average evaluation metrics after each round and use the Wilcoxon signed-rank test to estimate whether the differences in the resulting 25 values of each metric are significant or not. Even though the paired t-test should not be used for this, we perform it to see if its *p* values would be different from those of the Wilcoxon test. Finally, we divide our third data set patient-wise into train and test sets so that the test set has 191 slices (19.9% of the total data), and compare the two U-Nets for binary segmentation. We use the Shapiro–Wilk test to test the normality of Dice and IoU values of different segmentation masks, t-test and Wilcoxon test to estimate their differences, and F-test, Bartlett’s test and Levene’s test to check if there are significant differences in variances.

### Results

The results of the binary classification task are summarized in the contingency table of Table 3 and the resulting values of the evaluation metrics are in Table 4. According two McNemar’s test computed from Table 3 separately for sensitivity among COVID-19 patients and specificity negative patients, the modified U-Net produced significantly higher sensitivity (*p* value < 5.07e−5) but significantly lower specificity (*p* value < 0.0207). The ROC curves of the modified U-Net and InceptionV3 can be seen from Fig. 1 and, according the DeLong test, there is no significant difference in their AUC (*p* value = 0.137).

**Table 3:** The contingency tables for comparing the performance of the modified U-Net and InceptionV3 in binary classification among both COVID-19 and negative X-rays separately.

	COVID-19		Neg.	
U-Net$\backslash$InceptionV3	Pos.	Neg.	Pos.	Neg.
Pos.	207	54	63	44
Neg.	19	20	24	169

**Table 4:** The evaluation metrics computed for the modified U-Net and InceptionV3 from Table 3.

CNN$\backslash$Metric	Acc.	Sen.	Spe.	Pre.	Youden	F1	$\kappa$	MCC	AUC
U-Net	0.757	0.870	0.643	0.709	0.513	0.781	0.513	0.527	0.845
InceptionV3	0.732	0.753	0.710	0.722	0.463	0.463	0.464	0.737	0.821

The median values of the evaluation metrics are in Table 5 for the multi-class classification task. According to t-tests and Wilcoxon tests, the modified U-Net is significantly better than InceptionV3, regardless of which metric is used. The *p* value of the t-test for macro-average F1-score is 6.47e−4 and less than 2.38e−5 for all the other metrics and, similarly, the *p* value of the Wilcoxon test for macro-average F1-score is 0.00116 and less than 6.37e−5 for all the other metrics.

**Table 5:** The median values of the evaluation metrics computed for the modified U-Net and InceptionV3 during the rounds of the multi-class classification task with repeated fivefold cross-validation.

CNN$\backslash$Metric	Acc.	Sen.	Spe.	Pre.		Youden	F1		$\kappa$	MCC
				Mac.	Mic.		Mac.	Mic.		
U-Net	0.857	0.714	0.905	0.760	0.714	0.619	0.693	0.714	0.619	0.641
InceptionV3	0.801	0.602	0.867	0.589	0.602	0.469	0.591	0.602	0.469	0.473

The results obtained with micro-averaging (Mic.) and macro-averaging (Mac.) are here separately for precision and F1-score but both these methods give the same values for accuracy, sensitivity, specificity, and Youden’s index.

The median and standard deviation of Dice and IoU values computed for the two U-Nets in the segmentation task are in Table 6, as are the *p* values of Shapiro–Wilk tests, t-tests, Wilcoxon tests, F-tests, Bartlett’s tests, and Levene’s tests. Based on these *p* values, neither Dice nor IoU values are normally distributed, the deeper U-Net is significantly better in terms of both Dice and IoU values, and, while the deeper U-Net had higher standard deviation, this difference is only significant according to Levene’s test performed for the IoU values.

**Table 6:** Median and standard deviation of Dice and IoU values computed from the binary segmentation masks predicted by two U-Nets for 191 image slices of the test set.

U-Net	Metric	Median	Std	Shapiro	t-test	Wilcoxon	F-test	Bartlett	Levene
64	Dice	0.484	0.300	1.11e−8	9.20e−5	1.52e−5	0.490	0.490	0.437
128	Dice	0.574	0.315	3.91e−10					
64	IoU	0.320	0.259	1.38e−7	1.42e−5	6.44e−5	0.182	0.182	0.0337
128	IoU	0.403	0.285	1.90e−8					

The U-Nets with different depths are distinguished here according to whether the maximum dimensionality of a Conv2D layer is 64 or 128. The table also contains the *p* values of Shapiro–Wilk tests for testing the normality separately for Dice and IoU values of each U-Net, and *p* values of t-tests, Wilcoxon tests, F-tests, Bartlett’s tests, and Levene’s tests comparing the two U-Nets.

## Discussion

In our first experiment, we used both McNemar’s test and the DeLong test to study two CNNs used for binary classification. Our results show that the choice of the threshold was not ideal for the modified U-Net as we obtained high sensitivity on the cost of the specificity. This also reveals one issue with McNemar’s test: It does not tell us which classifier is better if one of them has a significantly higher sensitivity but a significantly lower specificity. We would need to use some other thresholds to convert the output of the CNN into binary labels and then repeat McNemar’s tests in order to find out if the significant differences are caused by specific threshold choices or not. In this respect, the DeLong test is more useful as its results do not depend on the threshold choices. However, to obtain more trustworthy results, it would still be necessary to use cross-validation and compare the AUCs of different test sets with the Wilcoxon signed-rank test.

In our second and third experiments, we used the t-test for comparing the values of evaluation metrics, even though it is not recommend for this, especially not when combined with repeated cross-validation. Its *p* values were relatively close to those of the Wilcoxon tests and, regardless of which test was used, we obtained the same conclusions about the significant differences. Since the misuse of the t-test is rather common, as noted by Demšar^4^, it is good to know that the results obtained in earlier research are not necessary wrong. Similarly, even though the F-test is not designed for non-normally distributed data, its *p* values were very close to those of Bartlett’s tests. However, both the t-test and the F-test are sensitive to the error caused by potential outliers so their use can lead incorrect results.

It should be noted here that aim of our experiments was to give examples of the use of the evaluation metrics and the related tests. To find out how often the t-test or some other test produces false conclusions when improperly used, more research is needed. Similarly, one possible topic for future research is also how many the number of the test sets affects the trustworthiness of the conclusions.

## Conclusion

In this paper, we introduced several evaluation metrics for common ML tasks including binary and multi-class classification, regression, image segmentation, and object detection. Statistical testing can be used to estimate whether the different values in these metrics between two or more models are caused by actual differences between the models. The choice of the exact test depends the task of the models, the evaluation metric used, and the number of test sets available. As some metrics produce only one value from a single test set and there might be only one data set, some type of resampling, such as repeated cross-validation, is often necessary. Because of this, the well-known tests such the paired t-test underestimate variance and do not produce reliable results. Instead, the use of non-parametric tests such as the Wilcoxon signed-rank test or Friedman’s test is recommend.

## Data Availability

The X-ray data sets analyzed during the current study are available in the repositories: COVID-19 Radiography Database^61,62^ https://www.kaggle.com/datasets/tawsifurrahman/covid19-radiography-database, Chest X-Ray Images (Pneumonia)^63^ https://www.kaggle.com/datasets/paultimothymooney/chest-xray-pneumonia, and Tuberculosis (TB) Chest X-ray Database^64^ https://www.kaggle.com/datasets/tawsifurrahman/tuberculosis-tb-chest-xray-dataset.

## Code availability

Available at github.com/rklen/statistical_tests_for_CNNs.
