# Author Correction: Evaluation metrics and statistical tests for machine learning

**DOI:** 10.1038/s41598-024-66611-y

**Published:** 2024-07-08

**Authors:** Oona Rainio, Jarmo Teuho, Riku Klén

**Affiliations:** Turku PET Centre, University of Turku and Turku University Hospital, Turku, Finland

Correction to: *Scientific Reports* 10.1038/s41598-024-56706-x, published online 13 March 2024

The original version of this Article contained an error in the following equation in the Different machine learning tasks section, under the subheading ‘Multi-class classification’.

$${\text{T}}{{\text{N}}_i} = \sum\limits_{j \ne i} {{n_{jj}}}$$

now reads:

$${\text{T}}{{\text{N}}_i} = \sum\limits_{j \ne i} {\sum\limits_{h \ne i} {{n_{jh}}} }$$

The original Article has been corrected.

